# Fourteenth Annual Meeting of the Miss. Valley Asso. of Dental Surgeons,—Minutes

**Published:** 1858-03

**Authors:** 


### FOURTEENTH ANNUAL MEETING OF THE MISSISSIPPI VALLEY ASS. OF DENTAL SURGEONS.

### MINUTES.

The Association met, according to adjournment, in the Dental College, February 17th, 1858, at 10 o'clock, A. M.

Members present, Chas. Bonsall, James Taylor, J. P. Ulrey, W. R. Webster, G. W. Keely, H. A. Smith, J. Richardson, A. G. Stipher, H. R. Smith, E. Osmond, R. Peckover, J. Taft, and G. Watt.

The President and Vice President, being absent, Dr. C. Bonsall was elected president pro tem.

The Corresponding Secretary read the following letter from the President:

St. Louis, February 16, 1858.

*To Corresponding Secretary of Miss. Valley Association,*

Dear Sir:—It is with extreme regret that I find myself compelled to offer an apology for my absence from the annual meeting of the Association.

Until within a very short time it was my full intention to be present on this occasion; but circumstances which I can not control render my presence here necessary at this time, and I must submit to the disappointment with as good a grace as I can.

I sincerely trust and believe, that the gathering will be both an agreeable and a useful one; and I can not but repeat my regret in not being able to be present.

I enclose three dollars for yearly dues, and hoping to meet you all at the meeting of the convention in August next,

I remain, very truly, yours,

C. W. Spalding.

The Treasurer presented the following report:

Charles Bonsall Treasurer in account with Miss. Valley Association of Dental Surgeons.

| | | |
|---|---|---|
| Dr. 1857, Feb. 25, To Balance in the Treasury, $77 00. | | |
| " Initiation fees and dues since, 58 00. | | |
| Cr. 1857, Feb. 27, By Cash paid Janitor by order of Ass'n | $ 5 00 | |
| " " Reporter " | 10 00 | |
| 1858, Feb. 17, Balance in Treasury | 120 00 | |
| | | $135 00 |

On motion, Drs. Ulrey and Webster, were appointed a committee to audit the same, who reported as follows:

Your auditing committee find the Treasurer's account correct.

W. R. Webster,

J. P. Ulrey, } Committee.

The Committee on Membership, reported Dr. C. N. Woodward, of Ripley, Ohio, as a candidate for membership, who was unanimously elected.

The Treasurer reported that of those elected to membership at the last meeting, Drs. A. G. Philips, R. Walker, and

Chas. Silsbee had not yet paid their initiation fees, and had, consequently, failed to become members.

On motion of Dr. Taylor, it was *resolved*, that the name of no person be published in the list of members, till his initiation fee is paid.

Also, on motion of Dr. Taylor, it was *resolved*, that a list of the members be published in the minutes.

LIST OF MEMBERS.

Drs. James Taylor, Cincinnati, O.; Chas. Bonsall, Cincinnati, O.; A. Berry, Raymond, Miss.; J. P. Ulrey, Lawrenceburgh, Ia.; W. H. Goddard, Louisville, Ky.; Samuel Griffith, Louisville, Ky.; J. Taft, Xenia, O.; H. McCullum, Augusta, Ky.; J. W. Baxter, Warsaw, Ky.; Geo. Watt, Xenia, O.; J. G. Hamill, Xenia, O.; H. R. Smith, Cincinnati, O.; M. DeCamp, Mansfield, O.; W. R. Webster, Richmond, Ia.; A. S. Talbert, Lexington, Ky.; C. H. Quinlan, Chicago, Ill.; Geo. W. Keely, Oxford, O.; J. O. Martin, Franklin, Ia.; E. Osmond, Cincinnati, O.; A. G. Stipher, Decatur, Ill.; E. Bray, Evansville, Ia.; J. F. Mercer, Canada West; R. Peckover, Paris, Ky.; Jos. Richardson, Cincinnati, O.; D. W. Harris, West Liberty, O.; C. W. Spalding, St. Louis, Mo.; H. A. Smith, Oxford, O.; C. N. Woodward, Ripley, O.

The Executive Committee presented the following report:

The Executive Committee would respectfully present the following report on the order of business:

I. Report of Officers.

II. Report of Committees.

III. Addresses and Essays.

IV. Election of Officers.

V. Miscellaneous business, including as subjects for discussion:

1. What means will be most efficient in securing a healthy denture?

2. What is the most effectual treatment of exposed nerves, and what circumstances modify the treatment?

3. Filling Teeth.

4. What are the indications for the extraction of deciduous teeth?

5. Irregularity and its treatment.

6. To what extent may the atmospheric principle be applied to partial sets of teeth?

7. What are the merits and demerits of that method of inserting artificial teeth denominated, "*Cheoplasty*?"

J. TAFT,<br>

J. RICHARDSON, } Committee.<br>

J. P. ULREY.

The reading of essays was called for. Drs. Ulrey and Keely, the only essayists present, were at their own request excused.

Dr. Taylor offered the following, which was unamiously adopted.

*Resolved*, That, all the members of the dental and medical professions, who may be present during the different sessions of this Association be, and are hereby, invited to participate in the discussions.

The gentlemen included under this resolution are, Drs. King, Blakesly, Blandy, Wilson, Hamlin, Bracey, Horton, Leslie, Dills, Robinson, Ross, Hardy, Wartman, Wardle, Knowlton, Forbes, Awl, Daughtry, VanEmon, Barrere, Loud, Darling, Hunter, Budd, Wood, Chapman, and Roudabush. Messrs. J. M. Brown, J. T. Toland, and Jas. Leslie, were also present during most of the sessions.

On motion of Dr. Taft, 3 o'clock, to-morrow, was fixed for examination of instruments, morbid specimens, etc., and 4, P. M., to-morrow, for the election of officers.

On motion of Dr. Watt, the plates of the prize essay, and all that pertains to said essay, were committed to the care of Drs. Richardson and Taft, as a special committee, with the instructions given a year ago to the secretary.

On motion, adjourned to 2 o'clock, P. M.

FIRST DAY—AFTERNOON SESSION.

Met according to adjournment. The minutes were read and adopted.

On motion of Dr. Taylor,

*Resolved*, That each member in his remarks be limited to ten minutes.

The Association now proceeded to the miscellaneous discussions. No. 1 was taken up, and the members generally participated in the discussion. During the discussion of No. 2, adjourned till 10 A. M. to-morrow.

SECOND DAY—MORNING SESSION.

Met according to adjournment. The minutes were read and adopted. The discussion of No. 2 was resumed. Proceeding to No. 3. On motion adjourned to meet at 2 P. M.

SECOND DAY—AFTERNOON SESSION.

Met according to adjournment. The minutes read and adopted. Resumed the discussion of No. 3.

It being now 3 o'clock, the president announced the exhibition and examination of instruments, specimens, etc., to be in order. Messrs. Moses Dolph and J. T. Toland, each exhibited a neat rosewood dental case; Dr. Leslie presented "pyroxylin from cotton," (gun cotton) as a material for filling fangs; Mr. A. Packham exhibited specimens of lozenges containing subphosphate of lime, proposing this as a pleasant mode of administering this medicine; Dr. Ulrey presented two plaster models of primary dentures, containing each six incisors; Dr. A. M. Leslie exhibited a specimen of gold foil, prepared by beating together irregular fragments of foil,—exhibiting the welding properties of this metal; Dr. Taft exhibited a specimen of Fairbanks' impression cup. A variety of morbid specimens and miscellaneous appliances were presented—many of them exceedingly interesting, which the secretary is unable properly to accredit. Among these, however, were six teeth, extracted from the mouth of a lady, by her own hands, at a single *standing*, in front of a mirror, in the office of Dr. Webster.

The report of the Committee on dental progress was called for, and was read by the chairman, Dr. Richardson, and, on motion, accepted and adopted. [See page 282, Vol. xi. of Dental Register.]

Proceeded to the election of officers which resulted as follows:

*President*, W. R. WEBSTER.
*Vice President*, G. W. KEELY.
*Corresponding Secretary*, J. TAFT.
*Recording Secretary*, G. WATT.
*Treasurer*, C. BONSALL.
*Executive Committee*, J. P. ULREY, C. N. WOODWARD, H. A. SMITH.
*Committee on Membership*, E. OSMOND, A. G. STIPHER, J. RICHARDSON.
*Committee on Dental Progress*, J. TAYLOR, H. A. SMITH, J. TAFT.

President Bonsall appointed Drs. Taylor and Taft, a committee to conduct the president elect to the chair. On taking the chair Dr. Webster returned his thanks to the Association for the honor conferred, and pledged his best efforts for the promotion of the cause for which we are assembled.

On motion adjourned till 9. A. M. to-morrow.

THIRD DAY—MORNING SESSION.

Met according to adjournment. The minutes were read and adopted.

Dr. Taft read a paper from Dr. O. Willson, describing a case of irregularity and malformation of the jaws, and requesting information in regard to the proper treatment. The paper was on motion, accepted.

On motion of H. R. Smith, it was *resolved*, that a standing committee of three on dental irregularity, be elected, to whom this paper, and all connected with the subject be refered, with instructions to report on this paper in the June number of the Dental Register. The election of this committee was made the order of the day for 12 M. to-day.

Dr. Taft read an interesting extract from a letter from Dr. Perrine, of New York, in reference to the use of soluble quartz, as a material for filling teeth.

Drs. J. Taylor, H. R. Smith, and C. Bonsall, were elected the committee on irregularity, in accordence with the above resolution.

Proceeded to the discussion of No. 4. Adjourned till 2 o'clock, P. M.

THIRD DAY—AFTERNOON SESSION.

Met according to adjournment. Minutes read and adopted. proceeded to the discussion of No. 7.

Dr. Taylor offered the following resolutions, which were adopted:

*Resolved*, That the thanks of this association be tendered to Dr. Blandy, for the exhibition of numerous Cheoplastic operations.

*Resolved*, That the Treasurer be authorized to pay for such wood cuts as may be necessary to carry out and illustrate such cases as may come before the Committee on Irregularity.

*Resolved*, That the Secretary be paid by the Treasurer, fifteen dollars for writing out a report of our discussions.

*Resolved*, That the Janitor be paid five dollars, by the Treasurer, for services rendered during the present meeting of this association.

*Resolved*, That when we adjourn, we adjourn to meet the American Dental Convention, in this City, the first Tuesday of August, 1858, at 8 o'clock, A. M.

Adjourned till 7 o'clock, this evening.

THIRD DAY—EVENING SESSION.

Met according to adjournment. Minutes read and adopted.

The evening was principally spent in the discussion of Nos. 5 and 6, of miscellaneous business.

On motion of Dr. Bonsall, it was *resolved*, that the annual meeting of the Association, convene in the Dental College, on Wednesday, February 23, 1859, at 10 o'clock, A. M.

On motion of Dr. Smith, the thanks of the Association were returned to Dr. Bonsall, president, pro tem., for the satisfactory manner in which he presided over the present meeting.

On motion adjourned tc meet in the Dental College, Tuesday, August 3, 1858, at 8 o'clock, A.M.

---

